# Appropriate patient instructions can reduce prostate motion

**DOI:** 10.1186/1748-717X-7-125

**Published:** 2012-08-01

**Authors:** Reinhold Graf, Dirk Boehmer, Jacek Nadobny, Volker Budach, Peter Wust

**Affiliations:** 1 Department of Radiation Oncology, Charité Universitätsmedizin Berlin, Campus Virchow-Klinikum, Augustenburger Platz 1, 13353, Berlin, Germany

**Keywords:** Patient instruction, Fiducial markers, Prostate motion

## Abstract

**Background:**

Interfraction prostate motion must be compensated by increased safety margins. If filling status of rectum and bladder is constant, motion should be reduced. We attempted to reduce interfraction motion errors by proper patient instruction.

**Method:**

In 38 patients pairs of radio-opaque fiducial markers were implanted prior to definitive radiotherapy. Patients were positioned either according to skin marks or infrared body marker. We measured prostate displacement, i.e. pelvic bones versus intraprostatic marker position, via ExacTrac (two orthogonal radiographies) in 1252 fractions. Systematic and random setup and displacement errors were determined and safety margins estimated.

**Results:**

In our study interfraction prostate displacement is < 1 mm in RL direction, and < 2 mm in AP and SI direction. Systematic errors are slightly below random errors (< 1.5 mm). Positioning according skin marks results in higher inaccuracies of ±1.5 – 2 mm in RL and ±2 – 2.5 mm in AP/SI direction.

**Conclusions:**

In case of appropriate patient instructions (constant organ filling) the positioning via bone fusion requires CTV-PTV margins of 2 mm in RL, 4 mm in AP, and 5 mm in SI direction. Studies without any description of patient instruction found much higher margins of > 1 cm in AP and SI direction.

## Introduction

The dose–response relationship between long-term PSA control and radiation dose in the prostate is beyond controversy and validated by numerous studies and analyses, among them several randomized trials [1,2]. A minimum dose of 72 Gy (conventional fractionation) is required [3], but higher doses are desirable and further increase the rate and duration of PSA control. However, further increasing the dose towards 80 Gy elevates the dose in parts of the rectum and might be associated with late rectal toxicity [4].

The radiation exposure of the normal tissues surrounding the prostate, in particular the rectum, is mainly determined by the CTV – PTV safety margins. These margins can be influenced by the positioning technique of the patient and all measures to cover the CTV with the prescribed dose as accurate as possible.

Two major sources of uncertainty have been identified. Firstly, setup errors describe the variation of bony landmarks relative to skin marks, utilizing either laser crosses or infrared body markers. These errors depend on the diligence of the patient positioning (using certain positioning devices), the setup-procedure and in particular of the patient´s habitus. Employing modern image guidance such as portal images [5], MV-CT [6] or cone-beam CT [7] in conjunction with bone fusion this kind of set-up error can be minimized. Secondly, prostate motion relative to the pelvic bones is the remaining and dominant error source with variations of the prostate position either between fractions (interfraction) or during the irradiation (intrafraction). In order to correct the isocentre with respect to these displacements intraprostatic implanted markers are used, in particular metallic markers (gold, titanium). Marker based corrections have shown in numerous studies (see discussion), that the uncertainties caused by the prostate movement might be significant requiring safety margins > 1 cm. These errors are severe obstacles to further escalate the dose and should be reduced. Therefore, most investigators recommend the use of intraprostatic markers to track the prostate in order to reduce safety margins.

Prostate position or displacement depend on organ filling or distension, in particular of the rectum and to a minor degree of the bladder. Theoretically, prostate displacement can be reduced and is possibly less important, if a constant and reproducible organ filling is maintained. A well-defined reference situation might be an empty rectum and a bladder filled with a given content of some hundred millilitres. Some authors mention this reference filling state, but often this patient-dependent factor is not specified. Elaborated examinations about the relationship between patient preparation and prostate displacement are missing to our knowledge, and marker implantation is recommended.

However under clinical conditions, marker implantation is not only an additional invasive procedure (with some additional risk), but also time-consuming and expensive. Therefore, the question is reasonable if prostate motion can be reduced (and to which extent) by appropriate patient education. In the present study interfractional prostate motion has been investigated for a patient group, which has been carefully trained to keep the organ filling constant.

## Patients and methods

From 2005 – 2010 we offered patients with prostate carcinoma a definitive radiotherapy with an additional marker-based image guidance. All patients were irradiated by intensity-modulated radiotherapy technique (IMRT) at the dedicated stereotactic linear accelerator Novalis^TM^ (Brainlab AG, Feldkirchen, Germany) adjusting the setup by the ExacTrac/Novalis Body (ETNB) X-ray positioning system. On a routine basis an automatic bone fusion of the two non-coplanar oblique isocentric (stereoscopic) kV X-ray images with the corresponding digitally reconstructed radiographs (DRR) was executed. After marker implantation the correct setup (reference position) with respect to the prostate is achieved by manual fusion of the four marker end points adjusting the ETNB radiographs with the DRR. The marker end points have been specified in the planning CT during contouring of target volumes and normal tissues.

A total of 38 patients gave their informed consent to the marker implantation. We used either two stranded pairs of inactive marker seeds (until 2007, Isocord^TM^, Eckert & Ziegler, Berlin, Germany) and later on two VisiCoil^TM^ gold markers (Radiomed Corp Tyngsboro, MA, USA) with a length of 3 cm. The implantation was performed during a laparoscopic pelvic lymph node exploration without additional burden or during a minimal-invasive procedure via puncture through the perineum in local anaesthesia employing ultrasound and X-ray guidance. No grade 2 or higher complications were observed during/after the marker implantations.

For every patient, the planning CT was acquired in treatment position (head and neck support, knee and feet support) after a special patient instruction and preparation. The objective of this programme is a constant and reproducible filling of rectum and bladder, i.e. a rectum as empty as possible and a bladder filled with approximately 200–300 ml fluid. In detail, information sheets were handed out to patients with instructions on how to avoid flatulent food, too many fibres, and to empty the rectum prior to the planning CT and every single radiotherapy fraction. In addition, prior to the planning CT an enema was applied. Patients are trained on how to fill the bladder prior to CT and radiotherapy by drinking and how to adapt the amount of fluid to the required volume (measured in the planning CT) depending on the actual volumes registered after every radiotherapy session in a special measuring container.

At first the patient is positioned in treatment position according to skin marks or infrared markers. Then the ETNB radiographs are shot, the automatic bone fusion is accomplished and the setup is corrected according to the skeleton. In a second step a manual fusion with respect to the gold markers head and bottom is conducted. The correcting vector describes the prostate motion (displacement relative to the pelvic bone). These prostate displacements in three axes (left-right, superior-inferior, anterior-posterior) were determined in 1252 fractions. In addition, we registered the correcting vectors either from the skin marks (270 fractions) or the infrared markers (247 fractions) to the internal markers for a subgroup of patients to get an impression of the setup errors arising from conventional positioning procedures.

The *systematic* error ∑ of the whole sample is the standard deviation of the means of displacements per patient. The *random* error σ is defined as standard deviation of all displacements of the whole sample. From the systematic and random errors the required safety margins can be estimated according to statistical considerations [8]. We used the formula 2.5 ∑ + 0.7 σ to assess the *margin*.

## Results

The recommendations to keep the filling of rectum and bladder constant were accepted by all patients. Most patients demanded an exact timing of the radiotherapy session to achieve the reference volume in the bladder. The bladder size measured in the planning CT was communicated to the patient. Some discomfort arose from delays of the irradiation procedure, which is under clinical conditions sometimes inevitable. In consequence, patients assessed the desired bladder volume as difficult to achieve. On the other hand, defecation (emptying of the rectum) was estimated as easier because of increased stool frequency caused by mucosa irritation in most patients.

Systematic and random translational errors for the different setup procedures with respect to the marker-based reference positions are listed in Table 1. The lowest rubric (bone fusion) shows the prostate motion relative to the pelvic bone. As to expect the displacements averaged over all 1252 samples are near to zero (0.15 in LR, -0.64 in SI, -0.18 in AP direction). In our study with careful patient instruction and efforts the derived CTV-PTV margins are < 2 mm in lateral, and < 5 mm in the other directions.

**Table 1:** Systematic and random translational errors for conventional positioning with skin marks (laser crosses), infrared body markers and bone fusion (via ExacTrac/Novalis Body X-ray system = ETNB) by using the marker-based prostate position as “correct” reference position

**Setup**	**LR error [mm]**	**SI error [mm]**	**AP error [mm]**
Skin marks			
Σ	1.56	2.46	1.87
σ	1.82	2.31	2.51
Margin	5.17	7.76	6.43
Infrared markers			
Σ	1.35	0.75	0.96
σ	1.73	1.10	1.71
Margin	4.59	2.64	3.60
Bone fusion (ETNB)			
Σ	0.53	1.38	1.10
σ	0.75	1.84	1.79
Margin	1.85	4.74	4.00

The recommended safety margins according to the formula of van Herk (margin = 2.5 ∑ + 0.7 σ, see text) along the three axes are given in the third line.

The positioning errors are clearly higher if the patients are aligned on the treatment table according to the laser crosses at the skin (see the upper rubric in Table 1). Here, CTV-PTV margins of up to 8 mm are required. Interestingly, margins by use of infrared body marker (middle rubric) are small and of similar magnitude as the margins caused by prostate motion, at least for the AP and SI directions. Errors to the lateral directions are, however, higher by using body markers and require margins of 5 mm.

## Discussion

Radiotherapy technology has further evolved during the last years. While the improvement of dose distributions by using IMRT is common since 1995 now the next technological step IGRT (image guided radiotherapy) is approaching routine use. IGRT basically increases the *therapeutic ratio* by reduction of the PTV, i.e. the CTV – PTV safety margin. In this way, either the surrounding tissues can be better spared or the dose in the prostate can be further escalated. However, no prospective randomized study has been conducted until now to validate that relevant clinical endpoints such as PSA-control and/or late toxicity are unequivocally improved by IGRT. Conversely, even some studies indicate that uncritical use of IGRT can impair PSA control. Heemsbergen et al. 2007 [9] found a loss of PSA control of the patient group with higher rectum extension during planning CT, but they had reduced the PTV and relied on bone fusion without intraprostatic markers and/or appropriate patient preparation regarding rectum filling. Interestingly, the reduced PSA control was only seen in the subgroup of patients with high risk of seminal vesicles involvement. Engels et al. 2009 [10] described a negative prognostic impact of a distended rectum on PSA control even in case of marker-based image guiding techniques, but again without documented patient education. In contrast, for a sufficiently sophisticated prostate tracking via ultrasound the outcome did not depend on rectum filling during planning CT [11]. On summary, IGRT guarantees not automatically better clinical results and an elaborated analysis how to use IGRT is desirable.

It is evident that precision of prostate cancer radiotherapy can be enhanced by daily positioning control [12]. In order to reduce the geographic miss of the prostate fiducial intraprostatic markers are generally recommended. In several studies [8,13,14] large errors by interfractional prostate motion were found as summarized in Table 2. The LR displacements are consistently small, and the AP displacements are typically dominant. The SI displacements are in between, but can approach magnitudes near the AP movements. Authors explain the dominance of the AP direction with the variable filling of the rectum, and in fact sometimes found a bias of the ventral orientation. The variable rectum filling appears as major hindrance to further reduce the PTV.

**Table 2:** Systematic and random translational errors attributed to interfractional prostate motion (see Table 1) for a selection of studies (lines 2–4) in comparison to residual errors (line 5: estimated by the deviations between two measurement methods of prostate position) and errors by intrafractional prostate motion (line 6)

**Reference**	**Type of error**	**Left-Right (LR) [mm]**	**Sup-Inf (SI) [mm]**	**Ant-Post (AP) [mm]**	**Comments**
Current study Interfraction motion	∑= σ =	0.5 0.7	1.4 1.8	1.1 1.8	Bowel instructions
van Herk 2004 [8] Interfraction motion	∑ = σ =	0.9 0.9	1.7 1.7	2.7 2.7	Bowel instructions not mentioned
Soete et al. 2007 [13] Interfraction motion	∑ = σ =	1.3 1.6	4.2 2.3	4.3 2.8	Bowel instructions not mentioned
Tanyi et al. 2010 [14] Interfraction motion	∑= σ=	0,5 0,4	2,9 2,3	3,4 2,5	Bowel instructions not mentioned
Tanyi et al 2010 Residual error	∑= σ =	0.6 1.4	0.6 1.4	0.5 1.6	CBCT vs. Calypso
Tanyi et al 2010 Intrafraction motion	∑ = σ =	0.3 0.8	0.7 1.4	0.5 1.3	Calypso System

Σ = systematic error, σ = random error, LR = left-right, SI = superior-inferior, AP = anterior-posterior.

If in marker-based studies displacements of prostate movements were investigated, different conclusions are possible. Most investigators recommend marker implantation to eliminate these errors caused by motion, but do not trouble much about the causes of these variations. In the present study we attempted to reduce prostate movements by keeping the organ filling as constant as possible by careful patient instructions – and to verify this reduction by measurements. Interestingly, patient instructions regarding the bowel are not mentioned in the other studies listed in Table 2 (right row) even though this appears to warrant additional accuracy during radiotherapy administration.

Our comparisons in Table 2 and 3 indicate that motion-induced errors might in fact be considerably reduced by reasonable patient instruction. In particular, the systematic errors in AP-direction are lowered by a factor of 2–4, and in consequence the CTV-PTV margins are more than halved. Smitsmans et al. 2008 found as well a trend of reduced interfraction prostate motion by a dietary protocol [15].

Therefore, instructions to empty the rectum and fill the bladder can at least partially replace marker based tracking. It is on the other hand probable, that routine use of intraprostatic markers can reduce setup errors, but would not completely prevent inaccuracies [10]. This has to be balanced with patient risk, patient burden and additional time, effort and costs – and therefore the overall benefit of marker based image guidance has to be considered with care.

One major concern is migration of markers. In several investigations inter-marker distances have been registered during a radiotherapy course [16]. The measured variations of intermarker-distances are compatible with zero. This is in agreement with a few checks we performed during our study by repetitive CT-scans.

Nevertheless, a residual error is still expected for marker-based localisation incorporating all remaining inaccuracies such as geometric/mechanic uncertainties, rotational errors and inaccuracies of the image processing. This residual error can be estimated if two different measurement methods are simultaneously adopted and compared such as marker-based cone-beam CT (CBCT) and a marker-based (transponder) electromagnetic localisation method (Calypso®) [13]. Tables 2 and 3 (lines 5) demonstrate that the residual errors and the derived margins are only slightly below the errors and margins of our study. The advantage is at maximum 2 mm in SI-direction (2.6 instead of 4.7 mm).

**Table 3:** Safety margins attributed to interfractional prostate motion (see Table 1) calculated with the van Herk formula (margin = 2.5 ∑ + 0.7 σ)

**Reference**	**Margin [mm] LR**	**Margin [mm] SI**	**Margin [mm] AP**
Current study Interfraction motion	1.8	4.7	4.0
van Herk 2004 [8] Interfraction motion	2.9	5.4	8.6
Soete et al 2007 [13] Interfraction motion	4.4	12.1	12.7
Tanyi et al 2010 [14] Interfraction motion	1.6	8.9	10.2
Tanyi et al 2010 Residual error	2.5	2.6	2.3
Tanyi et al 2010 Intrafraction motion	2.8	3.7	3.2

LR = left-right, SI = superior-inferior, AP = anterior-posterior.

The results of the current study are compared with other published data (Σ = systematic error, σ = ramdom error).

Rapid intrafractional movements are additional sources of errors, which are inevitabble and therefore a limiting factor. The errors and resulting margins (lines 6 in Tables 2–3) are even higher than the residual errors and approach the values in our study. Intrafraction motion of prostate has been investigated in various series [17], and the standard deviations are quite comparable ranging around 1 mm in LR-direction and between 1 – 3 mm in SI or AP direction. The derived safety margins are approximately 3 mm in all directions and appear as a lower threshold. By statistical reasons the most important error components, i.e. inter-fraction and intra-fraction motion, should be of comparable size.

Table 3 illustrates that margins induced by interfraction motion in conjunction with patient instructions (line 1) and intrafraction motion (line 6) are close together with 1 mm or less difference. Under these circumstances the inter-fraction motion is the dominant error source with a combined margin (estimated as the root of the quadratic sum) 3.3 mm in LR, 6.0 mm in SI, and 5.1 mm in AP direction. If we attempt to eliminate the inter-fraction motion error by marker implantation and marker-based localisation (CBCT or ExacTrac) a residual margin (line 5 in Table 3) will still remain and add to the intra-fraction motion error. Then the combined margin would be estimated as 3.8 mm in LR, 4.5 mm in SI, and 3.9 mm in AP direction. Therefore in our study the margin reduction caused by marker implantation is only in the range of one millimetre. This is in fact quite unspectacular and must be charged against the disadvantages of marker implantation. If however large variations of rectum content (lines 2–4, Table 3) are tolerated, the required margins are clearly too large, and marker-based image guidance appears inevitably.

Intrafraction motion can only be corrected by a marker-based on-line tracking system e.g. the Cyberknife. However this leads to a completely different radiotherapy schedule based on hypofractionation, which should primarily be investigated in studies. It should be noted that measures to reduce the interfraction motion will also reduce the intrafraction motion, because in the empty and relaxed intestine the peristalsis is reduced as well. Therefore, the suggested margins might be even too pessimistic.

## Conclusions

Careful patient instruction in order to achieve a constant anatomy (filling status of rectum and bladder) might be a successful strategy to permit reasonably low safety margins for IMRT/IGRT of prostate cancer. This procedure might be nearly as good as marker-based image guidance. Further marker-based studies are needed to specify the optimum strategy to minimize inter- and intrafraction movement and to clarify the role of IGRT technology to improve clinical outcome.

## Competing interests

There are no competing interests of the authors.

## Authors’ contributions

RG analysed the portal images, determined the errors, performed the statistical analysis and summarized the results in tables. PW initiated the study, treated the patients (together with RG), interpreted and compared the data with literature and drafted the manuscript. VB participated in designing the study and approved the treatment concepts. DB coordinated the recruitment of patients and data acquisition. JN performed treatment planning, preparation of data sets for IGRT as well as quality control and data analysis. All authors participated in the critical discussion of the data and their statistical analysis. All authors improved the manuscript and approved the final version.
